# Supplementary material for: Design and Nonadiabatic Photoisomerization Dynamics Study of a Three-Stroke Light-Driven Molecular Rotary Motor
Source: Int J Mol Sci. 2022 Mar 31;23(7):3908. doi: 10.3390/ijms23073908 (PMC8999534; doi:10.3390/ijms23073908)
Supplement: Supplementary file 1 [file ijms-23-03908-s001.zip › ijms-1634021-supplementary.pdf]

Supporting Information (SI) For:

Design and Nonadiabatic Photoisomerization Dynamics Study of a Three-Stroke Light Driven Molecular Rotary Motor

Jianzheng Ma[1], Sujie Yang[1], Di Zhao[1], Chenwei Jiang[1,*], Zhenggang Lan[2*], and Fuli Li[1]

[1]Ministry of Education Key Laboratory for Nonequilibrium Synthesis and Modulation of Condensed Matter, Shaanxi Province Key Laboratory of Quantum Information and Quantum Optoelectronic Devices, School of Physics, Xi'an Jiaotong University, Xi’an 710049, China

[2]SCNU Environmental Research Institute, Guangdong Provincial Key Laboratory of Chemical Pollution and Environmental Safety & MOE Key Laboratory of Environmental Theoretical Chemistry, South China Normal University, Guangzhou 510006, China; School of Environment, South China Normal University, Guangzhou 510006, China

*Corresponding authors. Email: jiangcw@xjtu.edu.cn (C.J.); zhenggang.lan@m.scnu.edu.cn (Z. L.).

Table of contents

1. Optimized geometries and potential energy surfaces of three-stroke LDMRM DDIY…………………………………………………….......S3

2. Optimized geometries and potential energy surfaces of Filatov's two-stroke LDMRM DTPN………………………………………........S8

3. Optimized geometries of conical intersection in the *EP-ZP* and *ZP-EM* photoisomerization processes of three-stroke LDMRM DDIY........S12

4. Time-dependent evolution of geometrical parameters in several typical trajectories of three-stroke LDMRM DDIY...................................S14

5. Cartesian coordinates for several structures of three-stroke LDMRM DDIY optimized with the OM2/MRCI method..........................S25

6. Cartesian coordinates for three isomers of Filatov's two-stroke LDMRM DTPN optimized with the CAM-B3LYP/6-31G(d) method ……………………………………………………………S32

7. References..............………………...S35

1. Optimized geometries and potential energy surfaces of three-stroke LDMRM DDIY

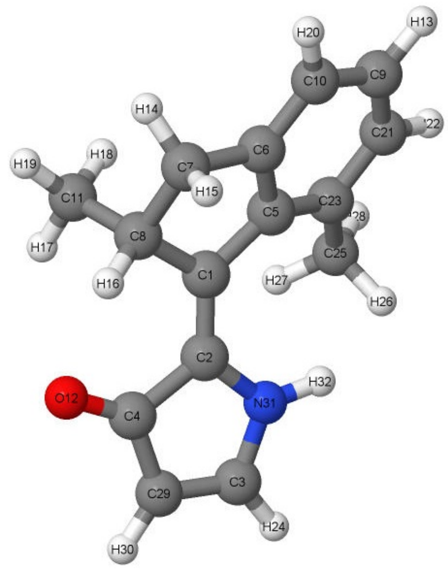

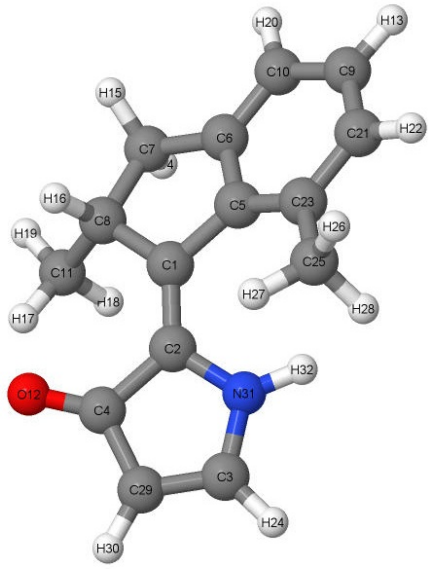

(a) (b)

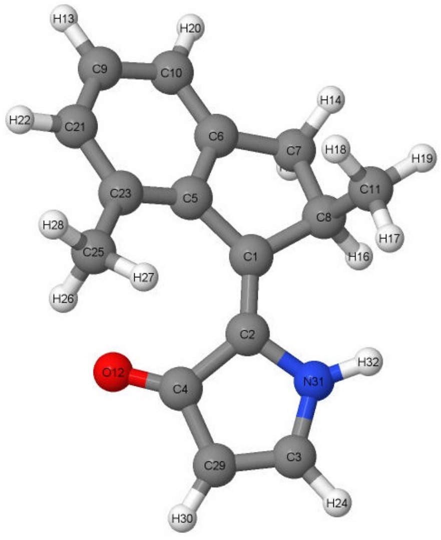

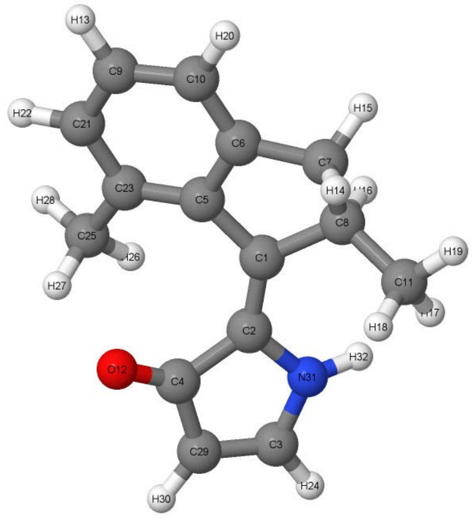

(c) (d)

Figure S1. Optimized geometries of (a) *ZP*, (b) *ZM*, (c) *EP* and (d) *EM* isomers calculated with OM2/MRCI method implemented in the development version of the MNDO program[S1].

Table S1. Optimized geometrical parameters of ground state three-stroke LDMRM DDIY, obtained from different methods. OM2/MRCI method is implemented in the development version of the MNDO program[S1].; while CAM-B3LYP/6-311G(d) and BH&HLYP/6-31G(d) method are implemented in Gaussian 09[S2] program. The lengths are in angstroms; the dihedral angles and bond angles are in degrees.

		CAM-B3LYP/ 6-31G(d)	BH&HLYP/ 6-31G(d)	OM2/MRCI
ZP	C1-C2	1.35	1.34	1.37
	C5-C1-C2	129.7	129.5	130.3
	C1-C2-C4	126.6	126.7	125.4
	C5-C1-C2-C4	178.4	178.3	181.4
	C5-C1-C2-N31	2.1	1.9	-1.2
	C2-N31-C4-C1	-1.5	-1.4	1.0
	C2-C1-C5-C23	32.8	33.5	32.1
ZM	C1-C2	1.35	1.35	1.37
	C5-C1-C2	125.3	125.3	126.5
	C1-C2-C4	131.0	130.9	129.6
	C5-C1-C2-C4	167.1	167.4	166.2
	C5-C1-C2-N31	-10.8	-10.6	-8.6
	C2-N31-C4-C1	-0.8	-0.8	-2.1
	C2-C1-C5-C23	-35.5	-35.6	-31.9
EP	C1-C2	1.35	1.34	1.37
	C5-C1-C2	131.1	131.1	130.2
	C1-C2-C4	131.2	131.1	131.9
	C5-C1-C2-C4	14.6	14.1	13.6
	C5-C1-C2-N31	-177.0	-177.5	-174.9
	C2-N31-C4-C1	4.6	4.6	3.4
	C2-C1-C5-C23	35.0	35.4	28.1
EM	C1-C2	1.35	1.35	1.37
	C5-C1-C2	127.9	128.0	127.3
	C1-C2-C4	129.2	129.2	129.9
	C5-C1-C2-C4	-18.0	-18.0	-19.9
	C5-C1-C2-N31	172.8	172.8	169.5
	C2-N31-C4-C1	-4.3	-4.3	-3.8
	C2-C1-C5-C23	-36.2	-36.1	-25.5

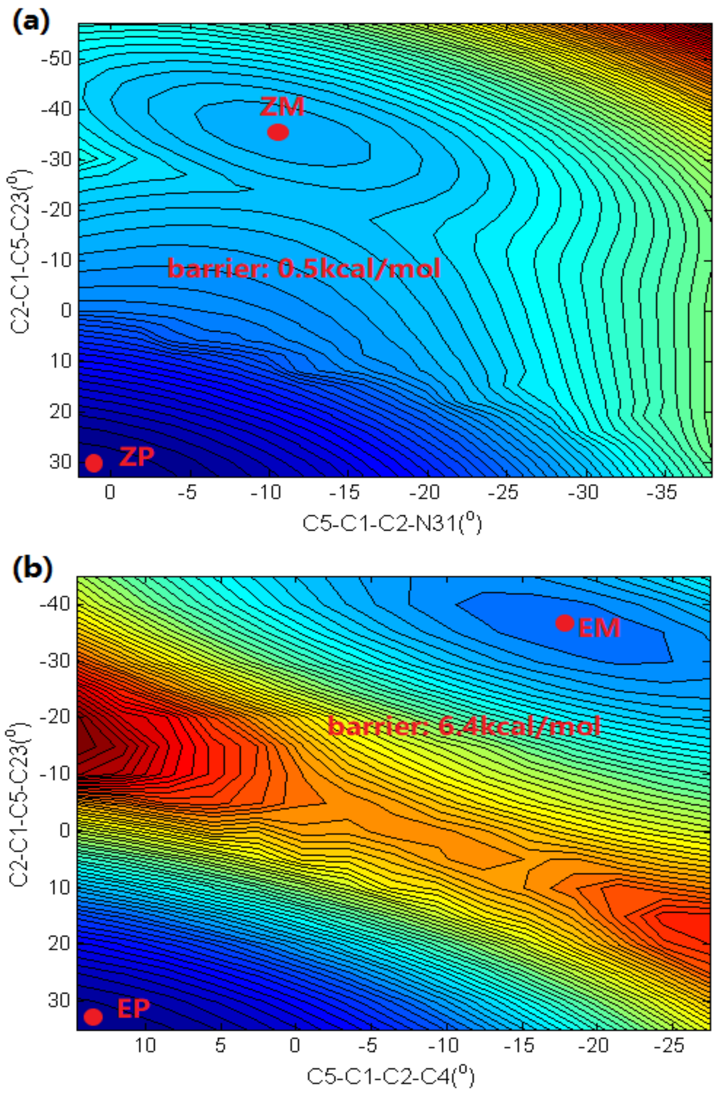

Figure S2. Two-dimensional contours of ground state potential energy surface of DDIY (a) near *ZP* and *ZM* geometries corresponding to dihedral angles C5-C1-C2-N31 and C2-C1-C5-C23; (b) near *EP* and *EM* geometries corresponding to dihedral angles C5-C1-C2-C4 and C2-C1-C5-C23. Corresponding potential energy surfaces were obtained with relaxed scan method at the CAM-B3LYP/6-31G(d) level, as implemented in Gaussian 09[S2] program. The energy barrier from *ZM* to *ZP* isomer, together with energy barrier from *EM* to *EP* isomer, are shown in subfigures (a) and (b), respectively.

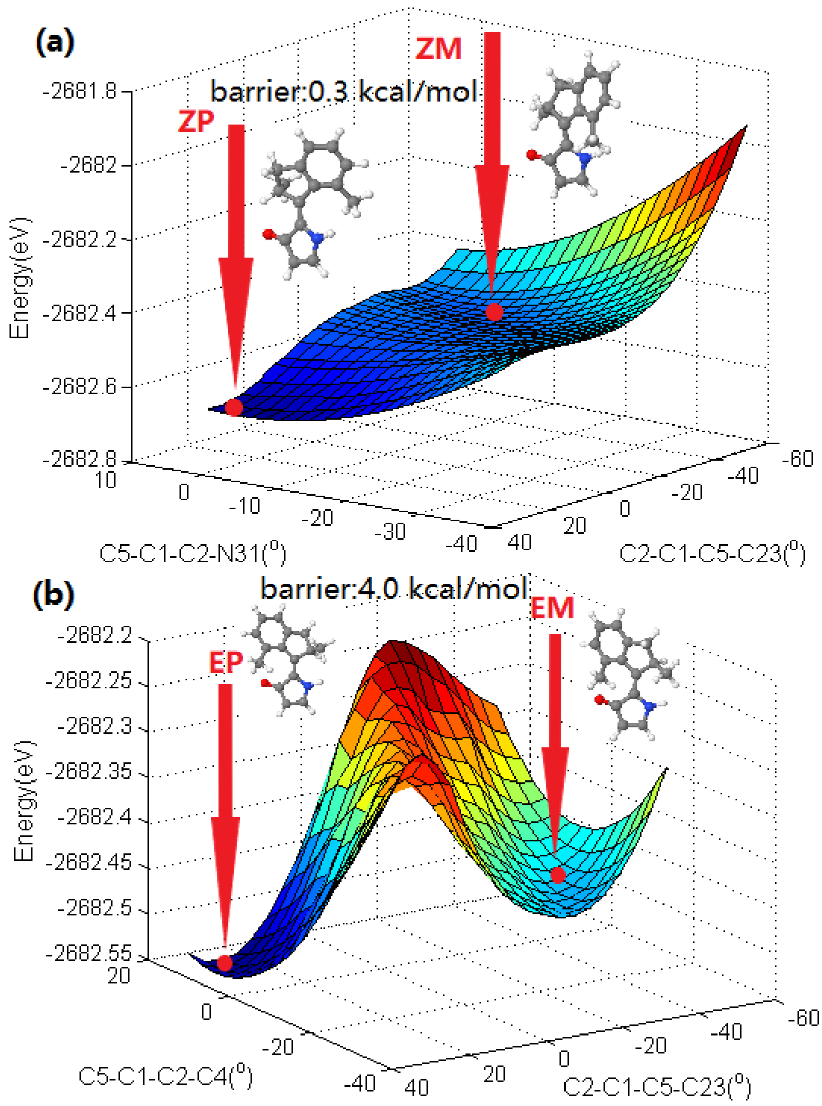

Figure S3. Ground state potential energy surface of DDIY (a) near *ZP* and *ZM* geometries corresponding to dihedral angles C5-C1-C2-N31 and C2-C1-C5-C23; (b) near *EP* and *EM* geometries corresponding to dihedral angles C5-C1-C2-C4 and C2-C1-C5-C23. Above potential energy surfaces were obtained with relaxed scan method at the OM2/MRCI level, as implemented in the development version of the MNDO program[S1]. The energy barrier from *ZM* to *ZP* isomer, together with energy barrier from *EM* to *EP* isomer, are shown in subfigures (a) and (b), respectively.

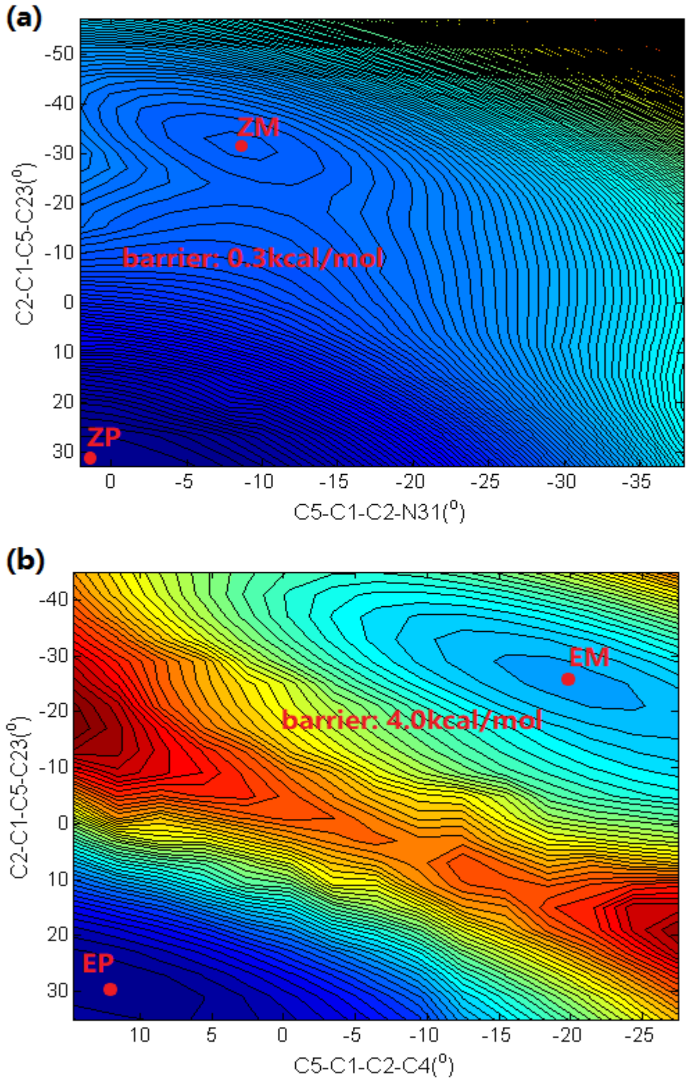

Figure S4. Two-dimensional contours of ground state potential energy surface of DDIY (a) near *ZP* and *ZM* geometries corresponding to dihedral angles C5-C1-C2-N31 and C2-C1-C5-C23; (b) near *EP* and *EM* geometries corresponding to dihedral angles C5-C1-C2-C4 and C2-C1-C5-C23. Corresponding potential energy surfaces were obtained with relaxed scan method at the OM2/MRCI level, as implemented in the development version of the MNDO program[S1]. The energy barrier from *ZM* to *ZP* isomer, together with energy barrier from *EM* to *EP* isomer, are shown in subfigures (a) and (b), respectively.

2. Optimized geometries of three isomers in Filatov's two-stroke LDMRM DTPN

Based on the CAM-B3LYP/6-31G(d) and BH&HLYP/6-31G(d) levels of theory, three local minima structures of Filatov's two-stroke LDMRM DTPN[S3] were obtained using Gaussian 09 program[S2], which are named as *EP*, *ZP* and *ZM* isomers, respectively.

The optimized geometries for three isomers of Filatov's two-stroke LDMRM DTPN[S3] calculated with CAM-B3LYP/6-31G(d) method are presented in Figure S2, while the corresponding geometrical parameters obtained with CAM-B3LYP/6-311G(d) and BH&HLYP/6-31G(d) methods implemented in Gaussian 09 program[S2] are summarized in Table S2. For comparison, optimized ground state geometrical parameters of *EP* and *ZP* isomers obtained by Filatov *et al.* [S3] are also presented.

The ground state potential energy surfaces near *EP* and *ZP* isomers obtained with relaxed scan method at CAM-B3LYP/6-311G(d) level are presented in Figure S3(a) and S3(b), respectively. As we can see, Figure S3(b) confirms the exsitence of *ZM* isomer of Filatov's two-stroke LDMRM DTPN. But the energy barrier from *ZM* to *ZP* isomer is only about 0.8 kcal/mol. Due to the low energy barrier and large kinetic energy of the molecules after nonadiabatic decay, photoisomerization trajectory starting from *EP* isomer can exceed the barrier and arrive at more stable *ZP* isomer at room temperature. Filatov *et al.* did observe direct *EP-ZP*

photoisomerization process, metastable *ZM* geometry was not observed during the working cycle of their LDMRM DTPN.

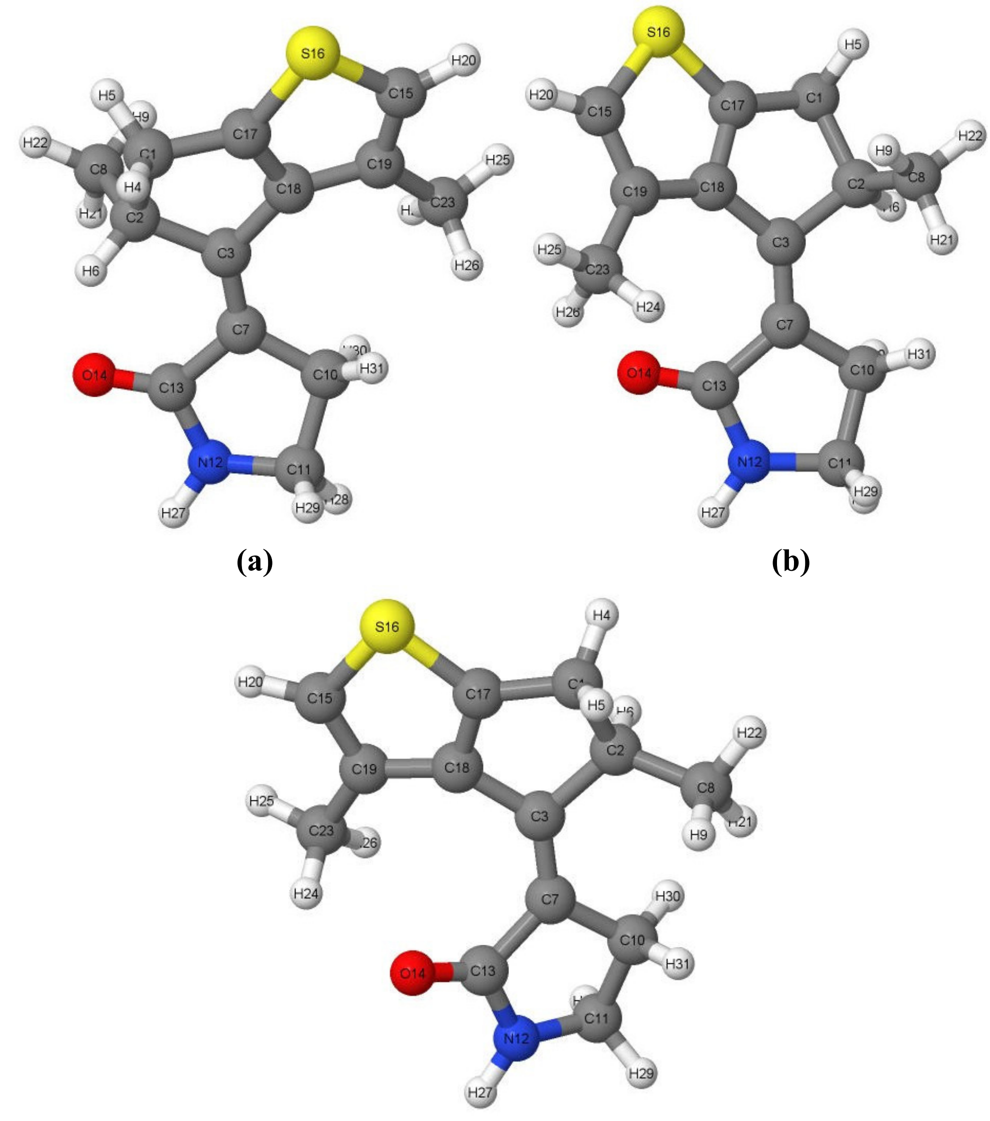

(a) (b)

(c)

Figure S5. Optimized geometries for three isomers of Filatov's two-stroke LDMRM DTPN[S3] calculated with CAM-B3LYP/6-31G(d) method implemented in Gaussian 09[S2] program. (a) *EP*, (b) *ZP* and (c) *ZM* isomers. All atoms are labeled.

Table S2. Optimized ground state geometrical parameters of Filatov's two-stroke LDMRM DTPN[S3], obtained from DFT methods. CAM-B3LYP/6-31G(d) and BH&HLYP/6-31G(d) methods are implemented in Gaussian 09 program[S2]. For comparison, optimized ground state geometrical parameters of *EP* and *ZP* isomers obtained by Filatov *et al.* are also presented. The lengths are in angstroms; the dihedral angles and bond angles are in degrees.

		CAM-B3LYP/ 6-31G(d)	BH&HLYP/ 6-31G(d)	SSR-BH&HLYP/ 6-31G(d)[S3]
EP	C3-C7	1.34	1.34	1.34
	C18-C3-C7	129.8	129.8	129.8
	C3-C7-C13	123.4	123.6	123.6
	C18-C3-C7-C13	177.4	177.3	177.3
	C18-C3-C7-C10	2.3	2.2	2.2
	C7-C10-C13-C3	-2.0	-2.0	-2.0
	C19-C18-C3-C7	22.1	22.5	22.7
ZP	C3-C7	1.34	1.34	1.34
	C18-C3-C7	133.1	133.1	133.3
	C3-C7-C13	127.9	127.9	128.0
	C18-C3-C7-C13	8.2	8.0	8.2
	C18-C3-C7-C10	-178.6	-178.7	-178.7
	C7-C10-C13-C3	2.8	2.7	2.8
	C19-C18-C3-C7	30.0	30.0	29.9
ZM	C3-C7	1.35	1.34	
	C18-C3-C7	129.7	129.9	
	C3-C7-C13	125.7	125.9	
	C18-C3-C7-C13	-15.2	-15.2	
	C18-C3-C7-C10	168.3	168.2	
	C7-C10-C13-C3	-1.4	-1.3	
	C19-C18-C3-C7	-28.0	-27.8	

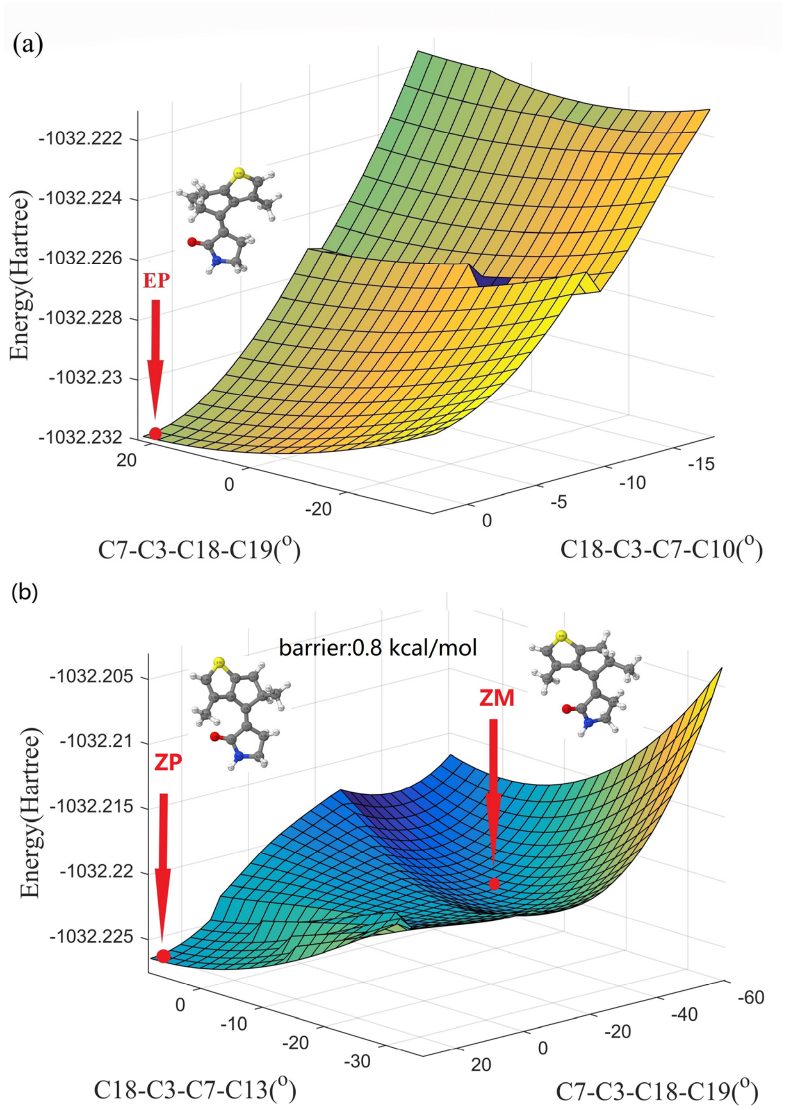

Figure S6. With dihedral angles (a) C7-C3-C18-C19 and C18-C3-C7-C10, (b) C7-C3-C18-C19 and C18-C3-C7-C13 as degrees of freedom respectively, the ground state potential energy surfaces near (a) *EP* and (b) *ZP* isomers of Filatov's two-stroke LDMRM DTPN[S3] were obtained from relaxed scan method at CAM-B3LYP/6-31G(d) level implemented in Gaussian 09[S2] program. The energy barrier from *ZM* to *ZP* isomer is also presented in the figure.

3. Optimized geometries of conical intersection in the *EP-ZP* and *ZP-EM* photoisomerization processes of three-stroke LDMRM DDIY

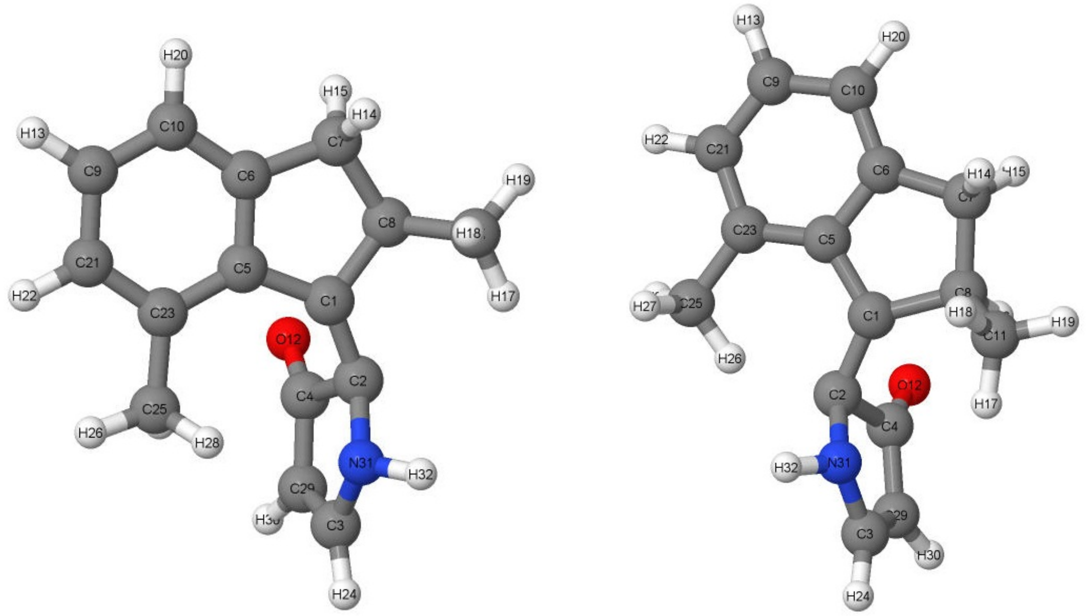

(a) **(b)**

Figure S7. Optimized geometries of two S_0/S_1 conical intersections (a) *ECI(1)* and (b) *ECI(2)* in the *EP-ZP* photoisomerization process calculated with OM2/MRCI method implemented in MNDO99[S1] program.

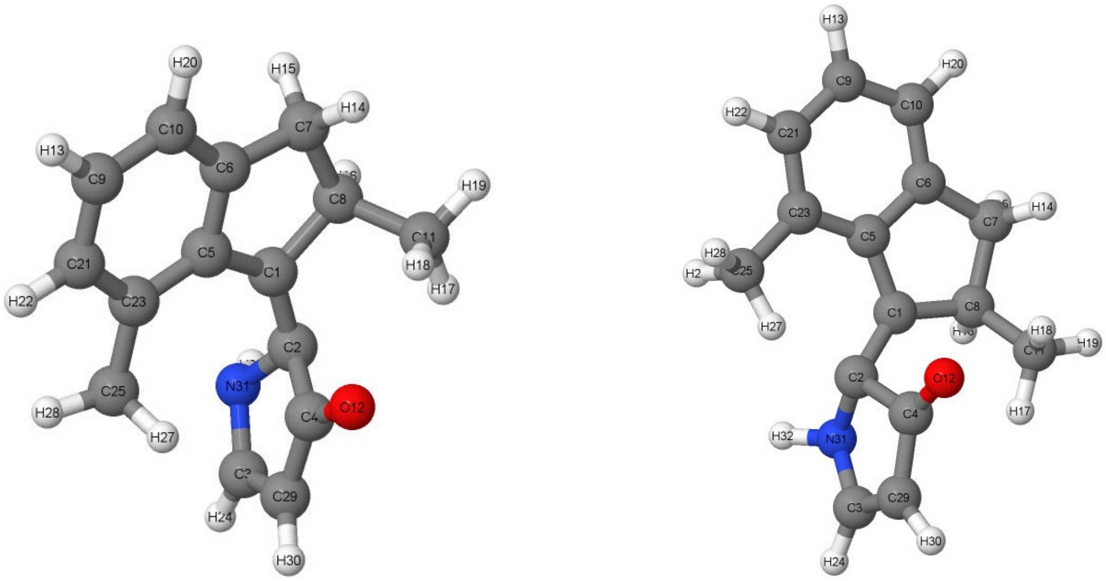

(a) **(b)**

Figure S8. Optimized geometries of two S_0/S_1 conical intersections (a) *ZCI(1)* and (b) *ZCI(2)* in the *ZP-EM* photoisomerization process calculated with OM2/MRCI method implemented in MNDO99[S1] program.

Table S3. Optimized geometrical parameters of two S_0/S_1 conical intersections *ECI(1)* and *ECI(2)* in the *EP-ZP* photoisomerization process calculated with OM2/MRCI method implemented in MNDO99[S1] program. The lengths are in angstroms; the dihedral angles and bond angles are in degrees.

		OM2/MRCI
ECI(1)	C1-C2	1.39
	C5-C1-C2	132.7
	C1-C2-C4	104.1
	C5-C1-C2-C4	55.8
	C5-C1-C2-N31	-67.9
	C2-N31-C4-C1	-25.8
	C2-C1-C5-C23	12.7
ECI(2)	C1-C2	1.42
	C5-C1-C2	122.0
	C1-C2-C4	104.2
	C5-C1-C2-C4	111.9
	C5-C1-C2-N31	-132.2
	C2-N31-C4-C1	31.1
	C2-C1-C5-C23	6.2

Table S4. Optimized geometrical parameters of two S_0/S_1 conical intersections *ZCI(1)* and *ZCI(2)* in the *ZP-EM* photoisomerization process calculated with OM2/MRCI method implemented in MNDO99[S1] program.. The lengths are in angstroms; the dihedral angles and bond angles are in degrees.

		OM2/MRCI
ZCI(1)	C1-C2	1.40
	C5-C1-C2	133.4
	C1-C2-C4	105.1
	C5-C1-C2-C4	-55.0
	C5-C1-C2-N31	66.4
	C2-N31-C4-C1	27.6
	C2-C1-C5-C23	-9.7
ZCI(2)	C1-C2	1.41
	C5-C1-C2	121.2
	C1-C2-C4	105.6
	C5-C1-C2-C4	-113.5
	C5-C1-C2-N31	129.7
	C2-N31-C4-C1	-30.8
	C2-C1-C5-C23	-6.1

4. Time-dependent evolution of geometrical parameters in several typical trajectories of three-stroke LDMRM DDIY

In order to explore the reaction dynamics of three-stroke LDMRM DDIY in detail, time dependent evolution of geometrical parameters in five typical trajectories for both *EP-ZP* and *ZP-EM* photoisomerization processes are presented in the following.

Time dependence of central bond length C1-C2, central dihedral angle C5−C1−C2−C4, central dihedral angle C5−C1−C2−N31, side dihedral angle C2−C1−C5−C23 and pyramid dihedral angle C2−N31−C4−C1 in five typical trajectories (named as trajectory 1-5, respectively) of *EP-ZP* photoisomerization process are shown in Figure S6-S10. The correpongding geometrical parameters in reaction product *ZP* isomer are also shown in the figures with red lines.

Time dependence of central bond length C1-C2, central dihedral angle C5−C1−C2−C4, central dihedral angle C5−C1−C2−N31, side dihedral angle C2−C1−C5−C23 and pyramid dihedral angle C2−N31−C4−C1 in five typical trajectories (named as trajectory 1-5, respectively) of *ZP-EM* photoisomerization process are shown in Figure S11-S15. The correpongding geometrical parameters in reaction product *EM* isomer are also shown in the figures with red lines.

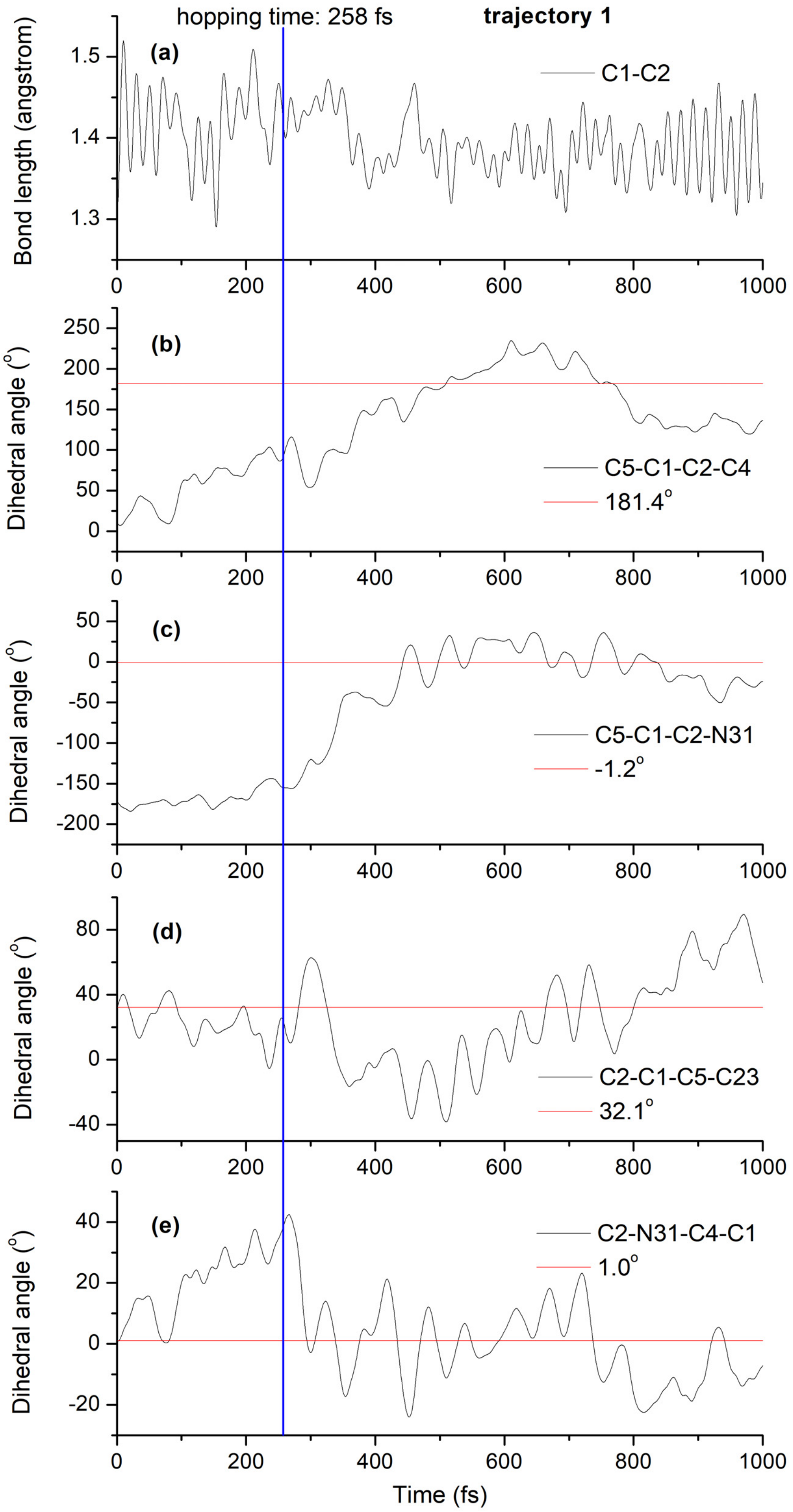

Figure S9. Time dependence of (a) central bond length C1-C2, (b) central dihedral angle C5−C1−C2−C4, (c) central dihedral angle C5−C1−C2−N31, (d) side dihedral angle C2−C1−C5−C23 and (e) pyramid dihedral angle C2−N31−C4−C1 in a representative trajectory (named as trajectory 1) of *EP-ZP* photoisomerization process.

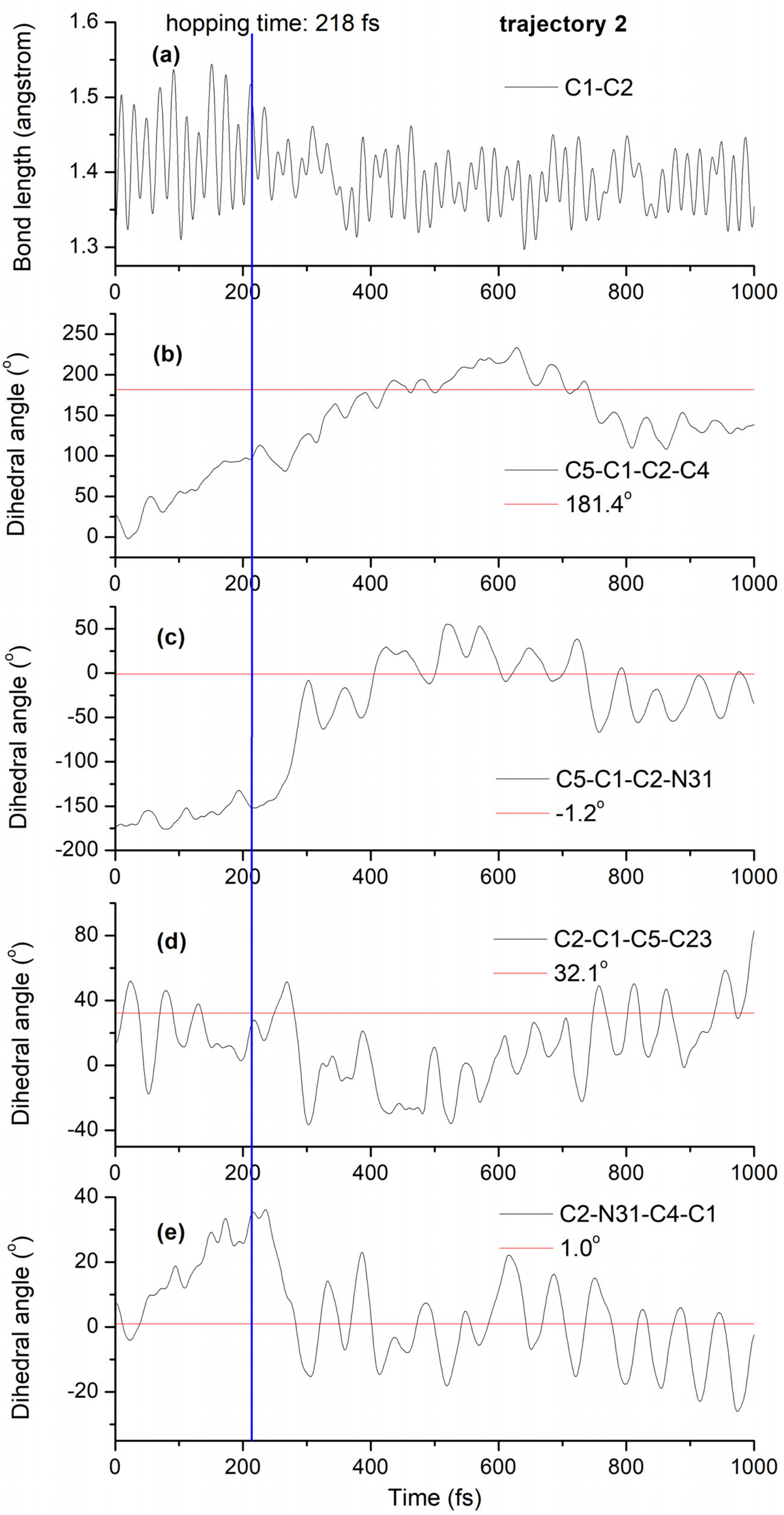

Figure S10. Time dependence of (a) central bond length C1-C2, (b) central dihedral angle C5−C1−C2−C4, (c) central dihedral angle C5−C1−C2−N31, (d) side dihedral angle C2−C1−C5−C23 and (e) pyramid dihedral angle C2−N31−C4−C1 in a representative trajectory (named as trajectory 2) of *EP-ZP* photoisomerization process.

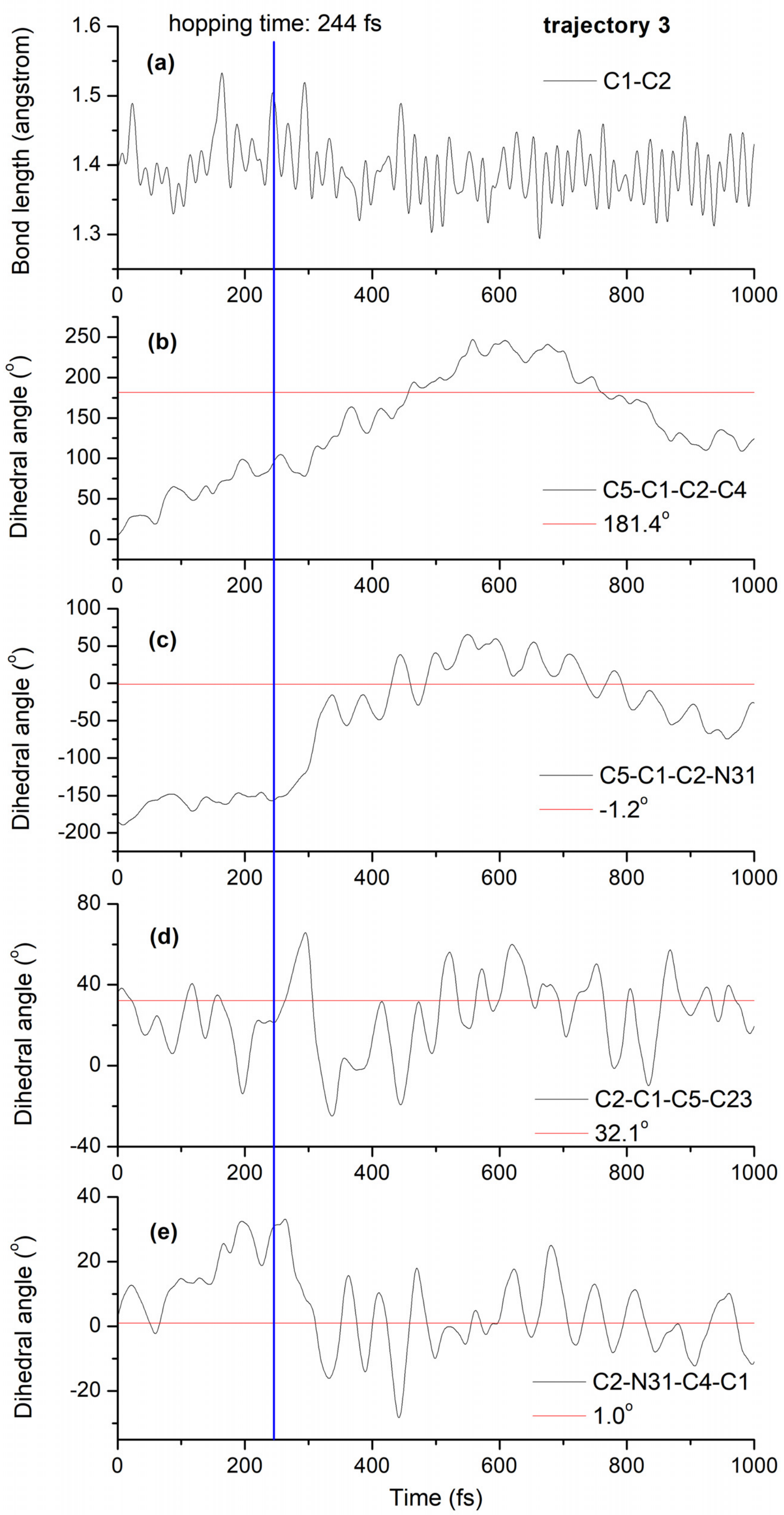

Figure S11. Time dependence of (a) central bond length C1-C2, (b) central dihedral angle C5−C1−C2−C4, (c) central dihedral angle C5−C1−C2−N31, (d) side dihedral angle C2−C1−C5−C23 and (e) pyramid dihedral angle C2−N31−C4−C1 in a representative trajectory (named as trajectory 3) of *EP-ZP* photoisomerization process.

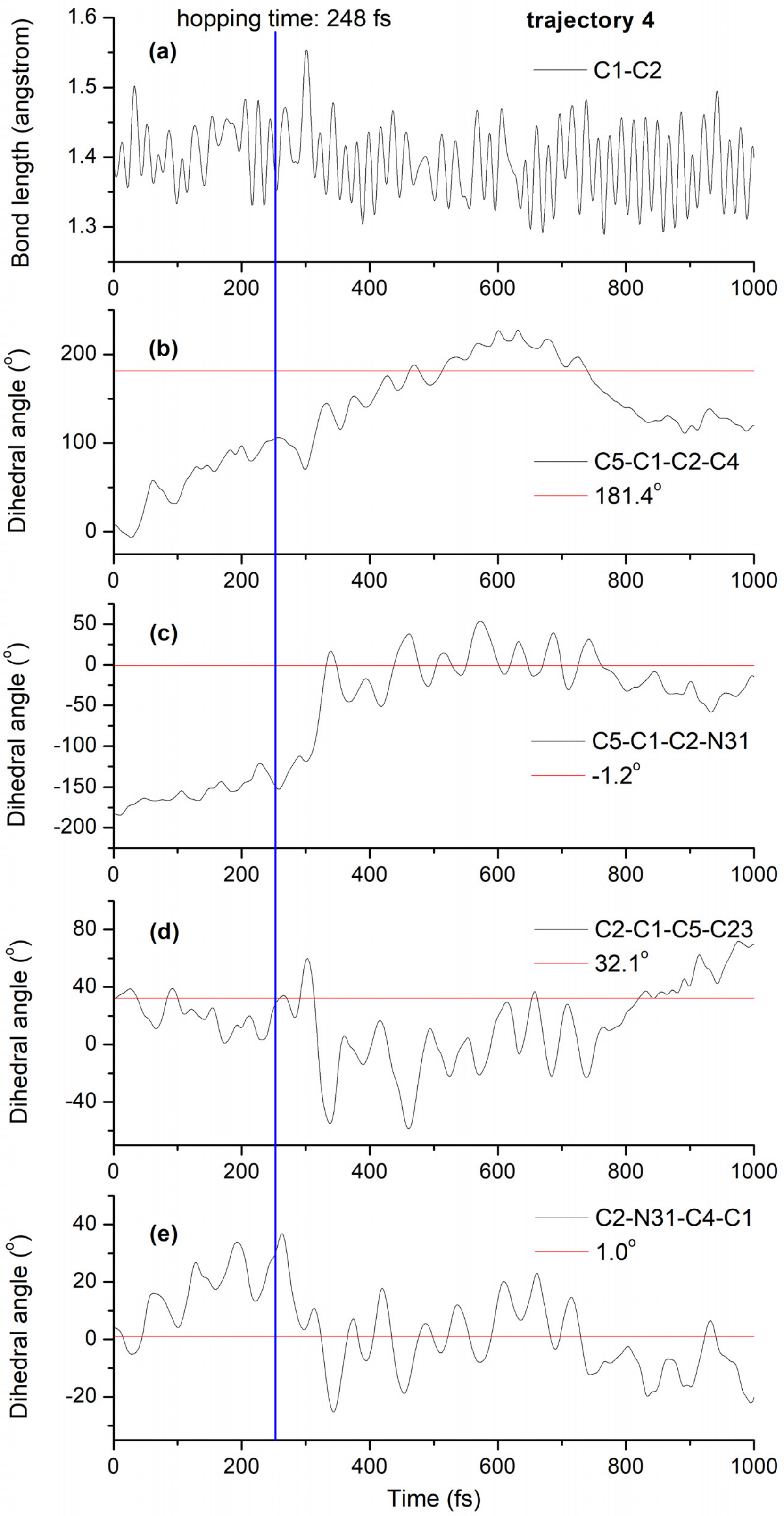

Figure S12. Time dependence of (a) central bond length C1-C2, (b) central dihedral angle C5−C1−C2−C4, (c) central dihedral angle C5−C1−C2−N31, (d) side dihedral angle C2−C1−C5−C23 and (e) pyramid dihedral angle C2−N31−C4−C1 in a representative trajectory (named as trajectory 4) of *EP-ZP* photoisomerization process.

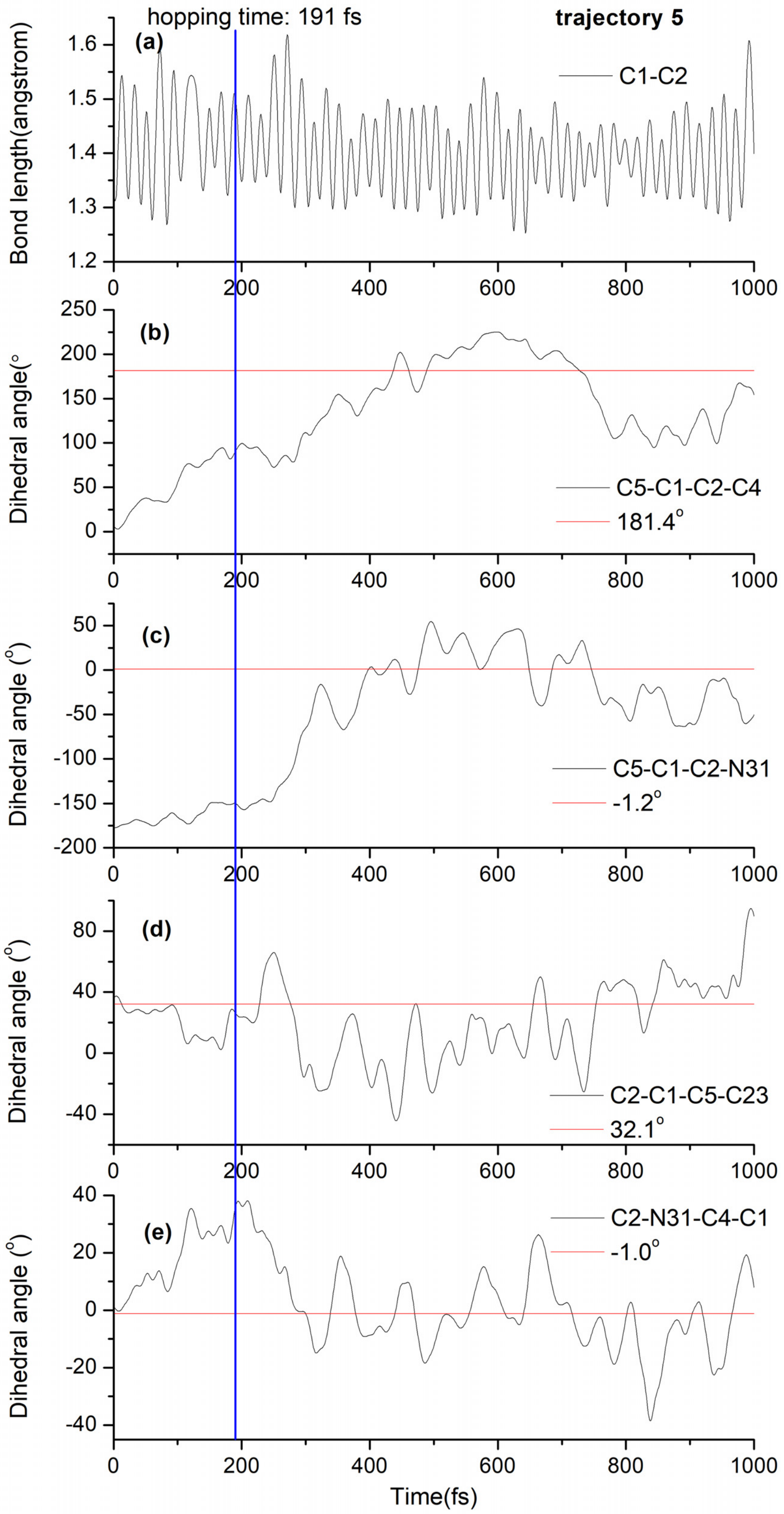

Figure S13. Time dependence of (a) central bond length C1-C2, (b) central dihedral angle C5−C1−C2−C4, (c) central dihedral angle C5−C1−C2−N31, (d) side dihedral angle C2−C1−C5−C23 and (e) pyramid dihedral angle C2−N31−C4−C1 in a representative trajectory (named as trajectory 5) of *EP-ZP* photoisomerization process.

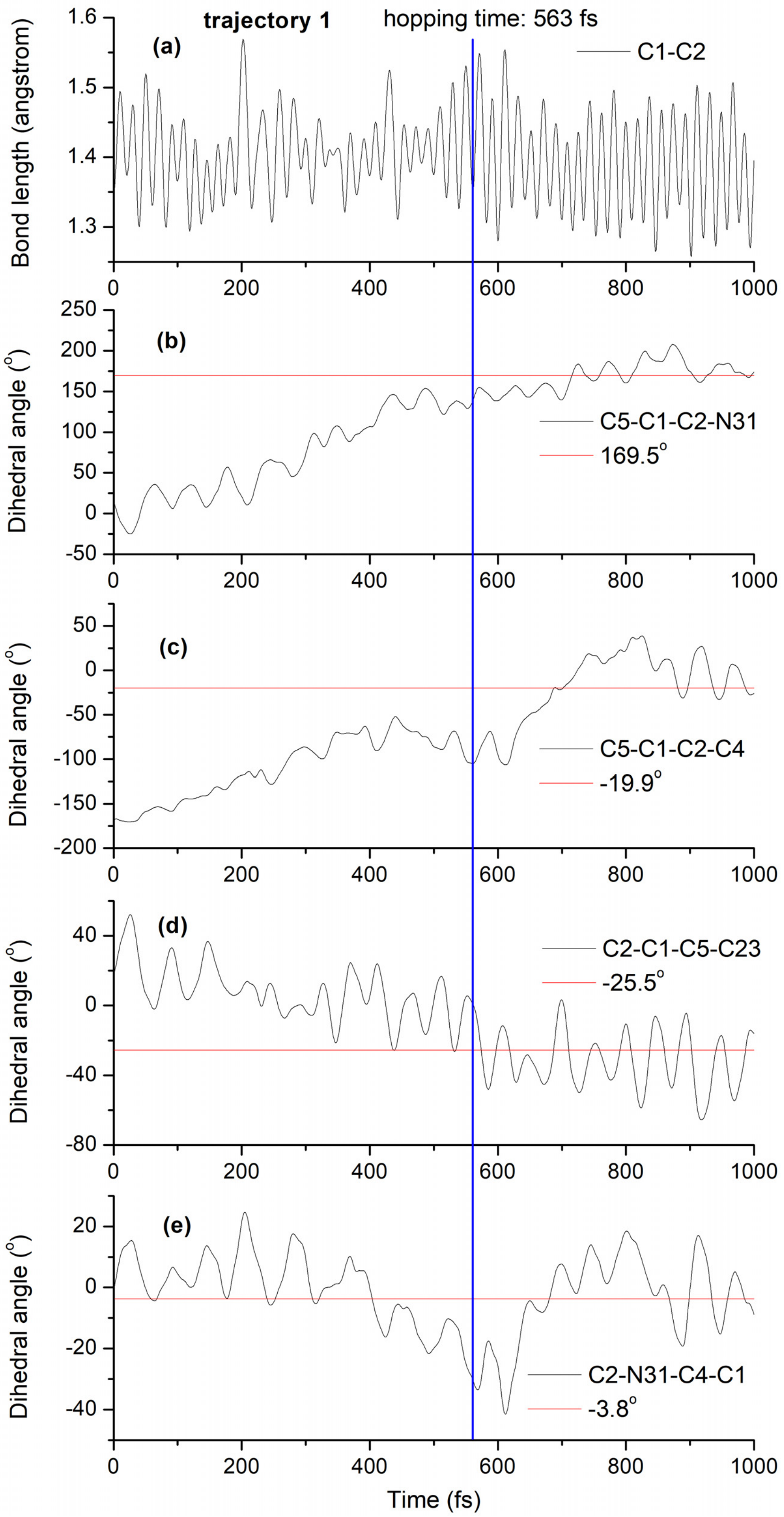

Figure S14. Time dependence of (a) central bond length C1-C2, (b) central dihedral angle C5−C1−C2−C4, (c) central dihedral angle C5−C1−C2−N31, (d) side dihedral angle C2−C1−C5−C23 and (e) pyramid dihedral angle C2−N31−C4−C1 in a representative trajectory (named as trajectory 1) of *ZP-EM* photoisomerization process.

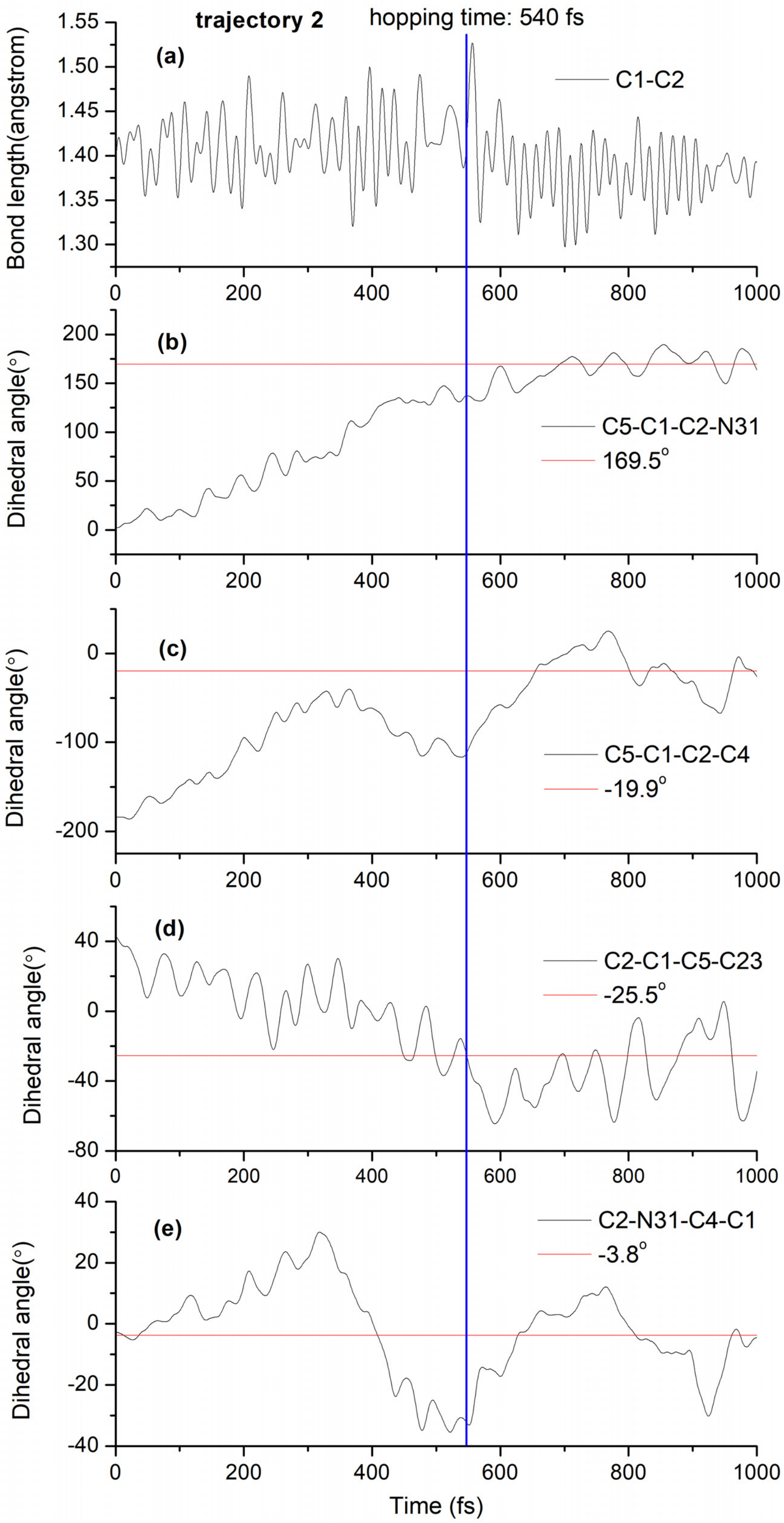

Figure S15. Time dependence of (a) central bond length C1-C2, (b) central dihedral angle C5−C1−C2−C4, (c) central dihedral angle C5−C1−C2−N31, (d) side dihedral angle C2−C1−C5−C23 and (e) pyramid dihedral angle C2−N31−C4−C1 in a representative trajectory (named as trajectory 2) of *ZP-EM* photoisomerization process.

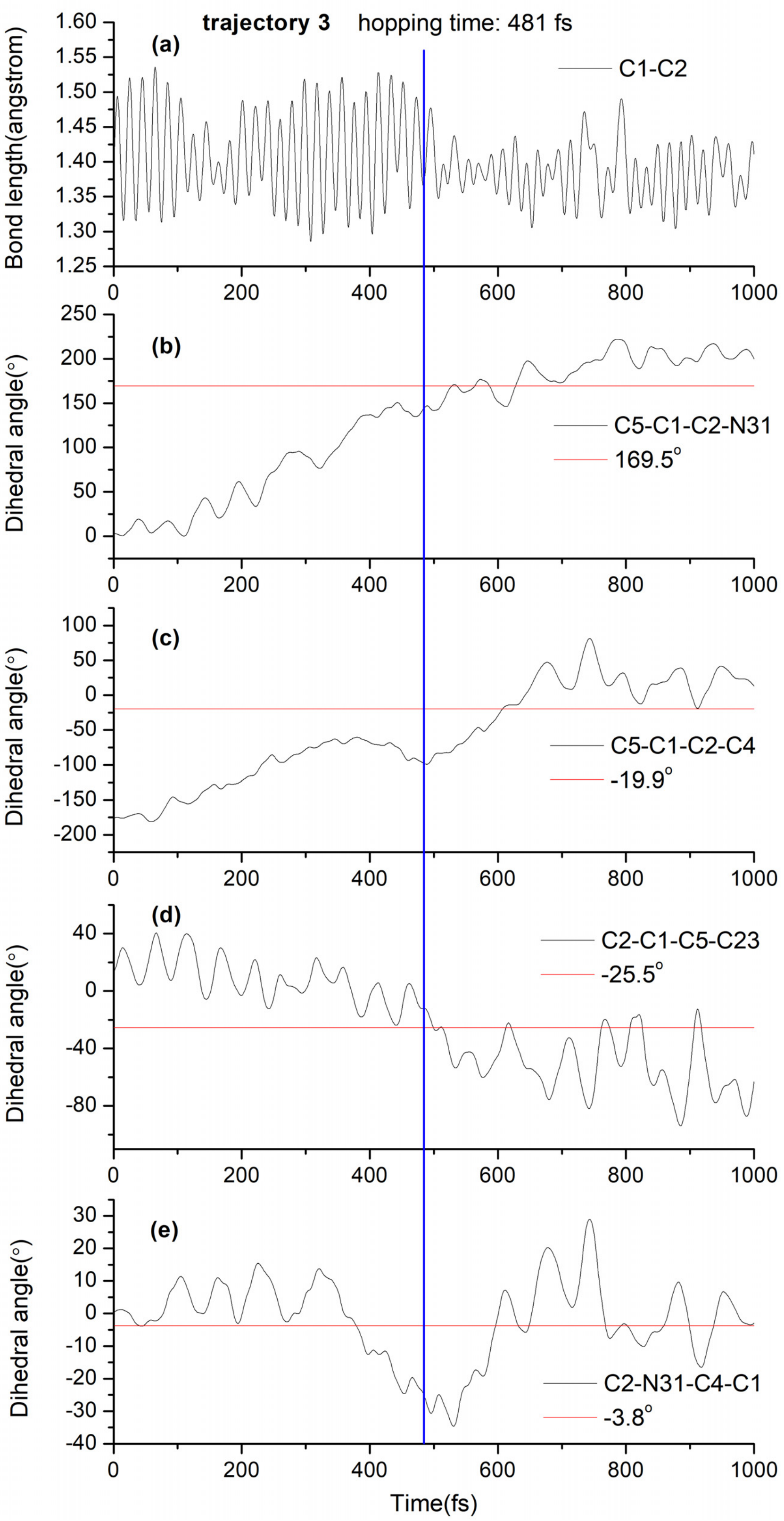

Figure S16. Time dependence of (a) central bond length C1-C2, (b) central dihedral angle C5−C1−C2−C4, (c) central dihedral angle C5−C1−C2−N31, (d) side dihedral angle C2−C1−C5−C23 and (e) pyramid dihedral angle C2−N31−C4−C1 in a representative trajectory (named as trajectory 3) of *ZP-EM* photoisomerization process.

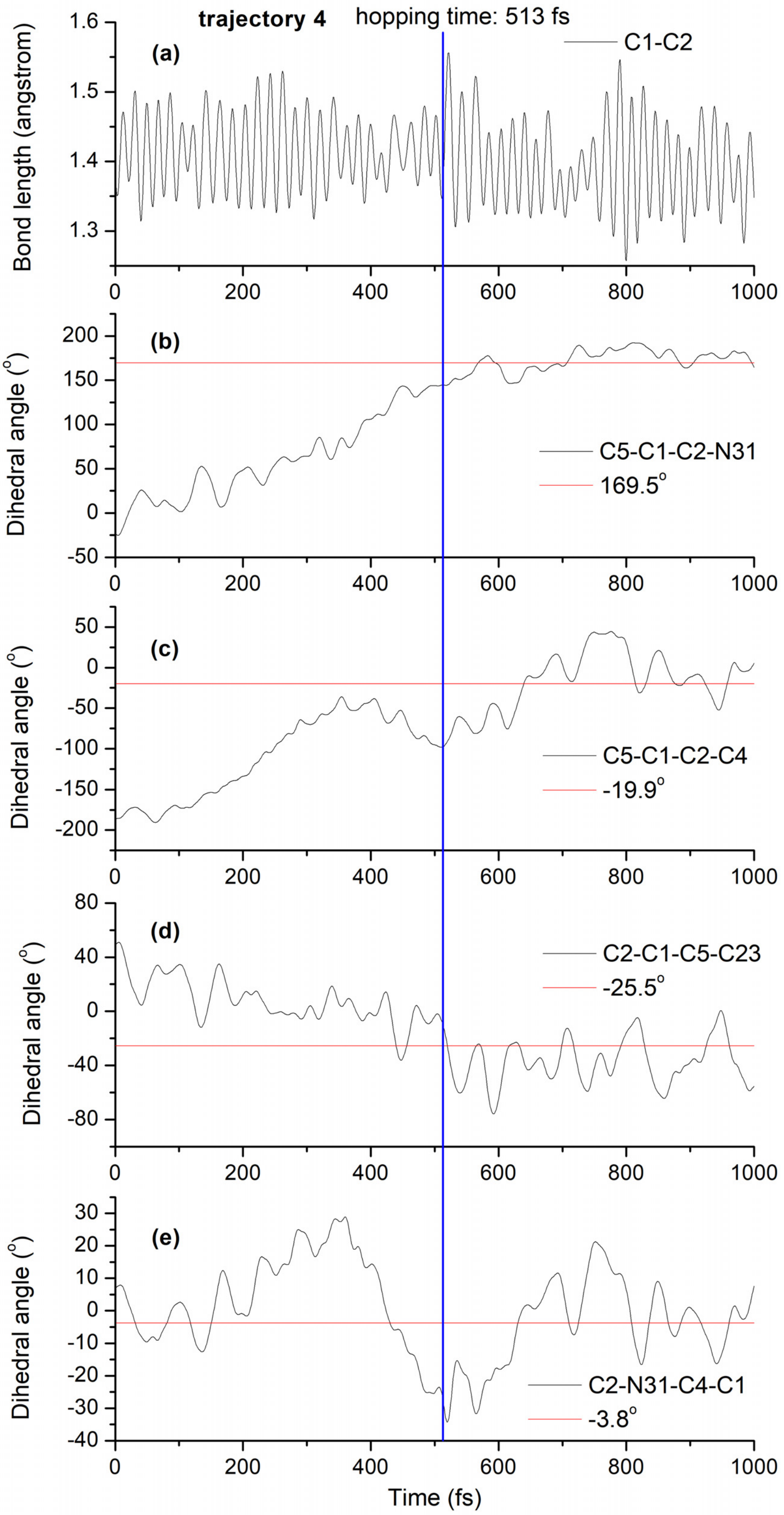

Figure S17. Time dependence of (a) central bond length C1-C2, (b) central dihedral angle C5−C1−C2−C4, (c) central dihedral angle C5−C1−C2−N31, (d) side dihedral angle C2−C1−C5−C23 and (e) pyramid dihedral angle C2−N31−C4−C1 in a representative trajectory (named as trajectory 4) of *ZP-EM* photoisomerization process.

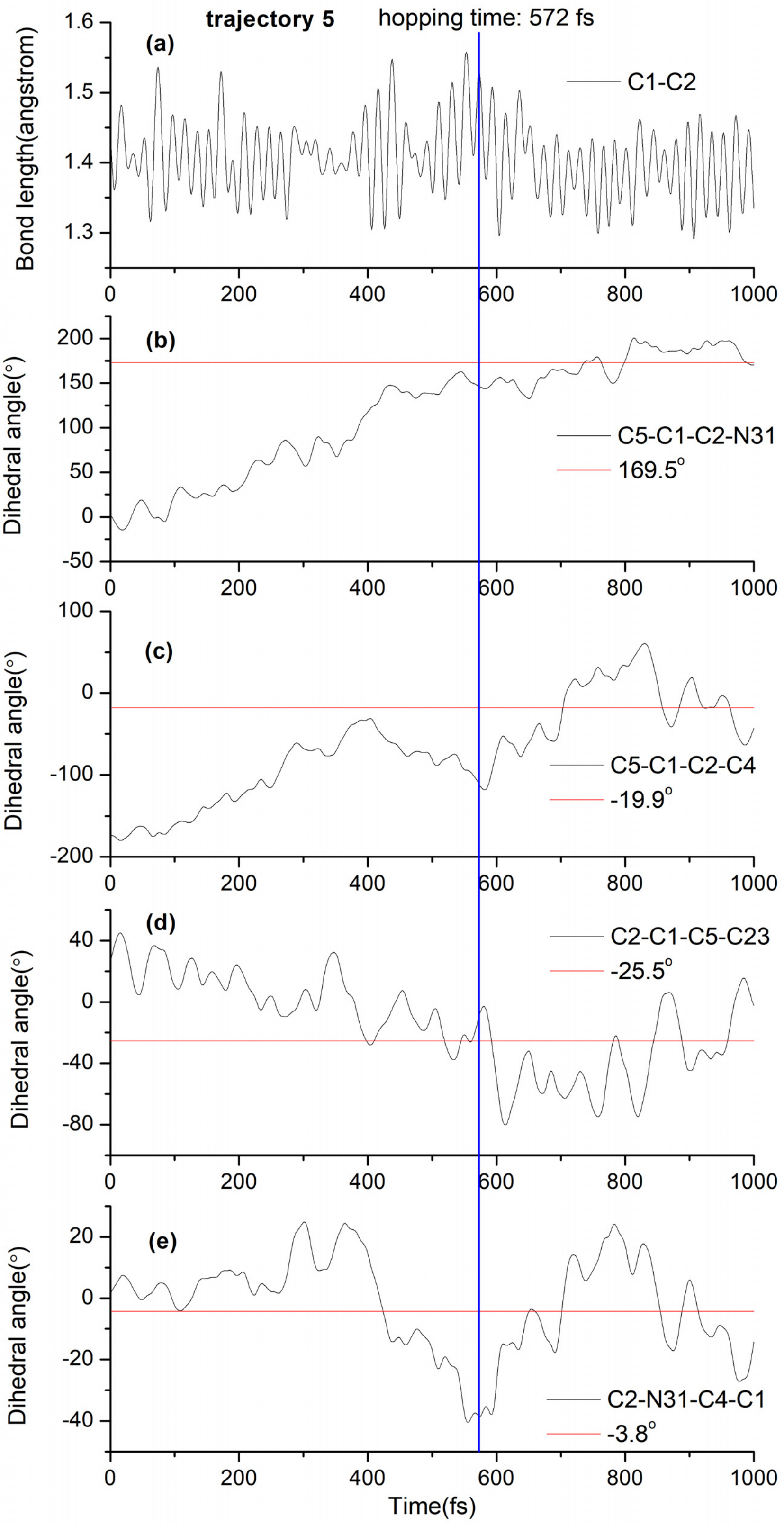

Figure S18. Time dependence of (a) central bond length C1-C2, (b) central dihedral angle C5−C1−C2−C4, (c) central dihedral angle C5−C1−C2−N31, (d) side dihedral angle C2−C1−C5−C23 and (e) pyramid dihedral angle C2−N31−C4−C1 in a representative trajectory (named as trajectory 5) of *ZP-EM* photoisomerization process.

5. Cartesian coordinates for several structures of three-stroke LDMRM DDIY optimized with the OM2/MRCI method

Unit of the Cartesian coordinate below is angstrom.

(1) *ZP* geometry at ground state.

C	0.2750443351	0.3700125800	-0.0202993675
C	1.4643832762	-0.2892385988	-0.1616219784
C	3.0425486914	-1.9186262367	-0.3649515743
C	2.8327543141	0.3508440060	-0.1109143091
C	-1.1051069040	-0.1444545098	-0.0241349823
C	-1.9374362037	0.9118537016	-0.4687985311
C	-1.1510166444	2.1694069165	-0.7074108653
C	0.2055790061	1.8862783101	-0.0325166523
C	-3.8667377742	-0.4925782290	-0.2880902994
C	-3.3100698777	0.7457410877	-0.6194041726
C	0.2306329987	2.3848234884	1.4015476089
O	3.0412751010	1.5569600595	0.0048942225
H	-4.9438450248	-0.6549901702	-0.4099749403
H	-1.6609248625	3.0463198481	-0.2601329244
H	-1.0279894232	2.3352229781	-1.7984445784
H	1.0495880083	2.3322868363	-0.6112757127
H	1.2003755053	2.1507413737	1.8594875823
H	-0.5701266943	1.9177906259	1.9957653868
H	0.0901060238	3.4782047684	1.4145932058
H	-3.9366521031	1.5625746604	-0.9855687437
C	-3.0710836506	-1.5088923384	0.2397328461
H	-3.5409925428	-2.4461343442	0.5620691117
C	-1.6818026782	-1.3522269077	0.3906798915
H	3.4369089335	-2.9331228467	-0.4555798625
C	-0.9204201047	-2.4447438961	1.0699273849
H	-0.6023779552	-3.2005242395	0.3294361101
H	-0.0489670590	-2.0349073144	1.5972573529
H	-1.5681162396	-2.9420203427	1.8083048076
C	3.7779040437	-0.7584313707	-0.2293558463
H	4.8459954022	-0.6577862495	-0.2342124817
N	1.6892707987	-1.6620573609	-0.3289078297
H	0.9743913779	-2.3674192789	-0.3950541704

(2) *ZM* geometry at ground state.

C	-0.2356184253	0.4332587176	0.1566298098
C	-1.3808045932	-0.2752903724	-0.1093054657
C	-2.7111300226	-2.0088287163	-0.7764354352
C	-2.8179428018	0.0606221415	0.2121306733
C	1.1491566252	-0.0722851925	0.0721839795
C	2.0034268936	1.0367817890	-0.1301524907
C	1.2279952308	2.3180348567	-0.1376188612
C	-0.1703138630	1.9308559184	0.3854984644
C	3.9164932383	-0.3981443472	-0.1681653626
C	3.3800416123	0.8884485499	-0.2647238664
C	-1.2349223591	2.7148002201	-0.3689254206
O	-3.2067169262	1.0569722464	0.8192718424
H	4.9936045342	-0.5517235712	-0.2995726330
H	1.1667436515	2.7120257640	-1.1740648679
H	1.7142899526	3.0752759709	0.5092083267
H	-0.2630070907	2.1278376066	1.4888006166
H	-2.2114672752	2.5590384279	0.0850858501
H	-1.2606368560	2.4039307344	-1.4249971832
H	-0.9875258055	3.7883530168	-0.3275158475
H	4.0228634916	1.7549111003	-0.4371126328
C	3.0981708356	-1.4826170058	0.1489388086
H	3.5531480076	-2.4653260029	0.3213574825
C	1.7062873120	-1.3396478556	0.2931417888
H	-2.9528349769	-2.9943545053	-1.1766180840
C	0.9369718993	-2.5197287800	0.7954695088
H	1.5501404153	-3.0609843789	1.5318158558
H	0.0115820298	-2.1996459468	1.2884001721
H	0.7055924370	-3.2121663087	-0.0323763496
C	-3.5945217203	-1.0958214796	-0.2367714406
H	-4.6611439916	-1.1807105089	-0.1657953059
N	-1.4136882842	-1.5597563253	-0.6744452722
H	-0.6097627966	-2.0541458807	-1.0243118814

(3) *EP* geometry at ground state.

C	-0.2522189976	0.6044405751	0.0383053531
C	-1.5454394664	0.1449868929	0.0579829789
C	-3.8282497139	0.2919406201	-0.0050040244

C	-2.1249760796	-1.2108117478	0.3535847348
C	1.0146220324	-0.1332392095	0.0426166042
C	2.0158152520	0.7498841581	0.5156489872
C	1.4523481242	2.1077552781	0.8133275625
C	0.0832551499	2.0815144563	0.1020724560
C	3.6610932978	-0.9613801476	0.2851390559
C	3.3348837763	0.3414160284	0.6771662965
C	0.1848916250	2.6580084735	-1.3011856187
O	-1.4969295498	-2.1927335761	0.7460799230
H	4.6979005372	-1.3067778392	0.3812352530
H	2.1041321788	2.9142084391	0.4225673091
H	1.3218395692	2.2360128440	1.9076773791
H	-0.6799998712	2.6477261921	0.6997881751
H	-0.7825930340	2.5787978066	-1.8222702391
H	0.9383569063	2.1067541019	-1.8785192593
H	0.4753009872	3.7190224817	-1.2551409080
H	4.0876531774	1.0110018288	1.0990812019
C	2.7033771904	-1.8021476932	-0.2784386878
H	3.0046323246	-2.7894352575	-0.6488862935
C	1.3566863931	-1.4089499708	-0.4267141740
H	-4.8065296685	0.7643881709	-0.1154485952
C	0.4063714827	-2.3178521257	-1.1216630598
H	-0.1447445774	-2.9007496594	-0.3795330997
H	-0.3090915296	-1.7333881889	-1.7209358677
H	0.9553267265	-2.9969115282	-1.7958557250
C	-3.5760493985	-1.0418611407	0.2507922109
H	-4.2928500586	-1.8233213930	0.4052575116
N	-2.6545159004	1.0126966174	-0.0565437766
H	-2.6065778604	1.9816556721	-0.3193283252

(4) *EM* geometry at ground state.

C	-0.2528633741	0.6315776011	0.2397745895
C	-1.4773437466	0.0175339273	0.1271773688
C	-3.7443394648	-0.2544042086	0.2433041293
C	-1.8411288924	-1.3041675450	-0.4973907603
C	1.0698168018	0.0015617782	0.1429024801
C	2.0173933359	1.0206492982	-0.1136258912
C	1.3586978646	2.3626814613	-0.1878944577
C	-0.0334405608	2.1205278681	0.4397982625
C	3.7821814366	-0.5817290139	-0.1285543224

C	3.3689180610	0.7457153600	-0.2844125322
C	-1.0647445784	3.0084708681	-0.2383390920
O	-1.0843847548	-2.0277586799	-1.1426791769
H	4.8444349783	-0.8310441452	-0.2432208565
H	1.2584830406	2.6750220428	-1.2484373600
H	1.9295382910	3.1310515067	0.3686311984
H	-0.0024015377	2.3433361446	1.5433241485
H	-1.9830085970	3.0984443080	0.3681609633
H	-1.3266483151	2.6051449061	-1.2244169012
H	-0.6513980314	4.0221604482	-0.3623796357
H	4.0800915686	1.5353168042	-0.5365536997
C	2.8730414553	-1.5769860525	0.2230136910
H	3.2372634079	-2.5925583905	0.4197478380
C	1.4967644080	-1.3133400992	0.3816196583
H	-4.7778591368	-0.0005745623	0.4866739138
C	0.6090445575	-2.4089925006	0.8546885945
H	-0.2119434924	-2.0035375124	1.4669276213
H	0.1866705490	-2.9267091025	-0.0119384575
H	1.1846214302	-3.1216275618	1.4693238600
C	-3.2937859643	-1.4169938271	-0.3467982816
H	-3.8762682130	-2.2601899431	-0.6603324825
N	-2.7063913289	0.6345917810	0.4379773588
H	-2.7940795737	1.4573616649	1.0076894021

(5) Four different S_1 /S_0 CIs for the three-stroke LDMRM DDIY.

ZCI(1)

C	0.2732282810	0.6018458157	-0.7314016296
C	1.6141843364	0.2082438660	-0.7532900973
C	3.0699455772	-1.5495358737	-0.6576926578
C	1.9769574727	-0.0382743507	0.6927659786
C	-0.9444008209	-0.0034750013	-0.1881775168
C	-2.0005386594	0.9422771598	-0.2588917041
C	-1.5509969324	2.2406458143	-0.8642419031
C	-0.0416052700	2.0483172450	-1.0640775747
C	-3.5052604849	-0.6391884783	0.7066462175
C	-3.2831253106	0.6365266619	0.1785652906
C	0.7782210042	2.9022237310	-0.1034312460
O	1.6354917810	0.6831617346	1.6474289053
H	-4.5081245919	-0.9031517765	1.0673024200
H	-1.7760265954	3.0773046686	-0.1781882845

H	-2.0717396511	2.4046172119	-1.8281929948
H	0.2706579387	2.2542314341	-2.1157131336
H	1.8379027770	2.6910423910	-0.2569051148
H	0.5223826714	2.6646756758	0.9345302931
H	0.5701008571	3.9657034997	-0.3061434432
H	-4.0904461537	1.3666787832	0.1174041719
C	-2.4797541297	-1.5814078593	0.7856072526
H	-2.6892783355	-2.5679839974	1.2088518288
C	-1.1793540311	-1.2876316293	0.3453548531
H	3.7634938994	-2.2886119557	-1.0422948786
C	-0.1049503529	-2.3105966011	0.4781791735
H	0.4534380858	-2.3940544812	-0.4561002346
H	0.5682776079	-1.9957466809	1.2796196938
H	-0.5380335052	-3.2917646685	0.7314270202
C	3.0208572950	-1.0609730400	0.6243067111
H	3.4916559108	-1.4716742572	1.5072585037
N	2.1330272903	-0.9275103158	-1.4691294377
H	2.4894515860	-0.6633139275	-2.3977022965

ZCI(2)

C	0.2537939023	0.2482710856	-0.5712551904
C	1.3568111731	-0.6276492260	-0.4488251291
C	3.5463098107	-0.7924017087	-1.1395022050
C	2.2694682336	0.0051239598	0.5931742396
C	-1.0691368349	-0.1529646620	-0.1556138898
C	-1.9441325600	0.9651108874	-0.1512754206
C	-1.2389613072	2.2067079463	-0.6147038743
C	0.2000667244	1.7415433399	-0.9033512025
C	-3.6893876821	-0.4270894034	0.6950990350
C	-3.2646248037	0.8333036061	0.2506328058
C	1.2235844860	2.5496934111	-0.1322425157
O	1.8917908945	0.4017474212	1.7059270914
H	-4.7264613535	-0.5452830552	1.0383294576
H	-1.2642279964	2.9655781774	0.1896320510
H	-1.7294096962	2.6129786828	-1.5206195637
H	0.4248929209	1.7938025450	-2.0018762264
H	2.2340756960	2.2054604223	-0.3819549097
H	1.0676858478	2.4326567549	0.9437740697
H	1.1301728178	3.6121368871	-0.4103704342
H	-3.9495398460	1.6813190317	0.2416036512

C	-2.8317595465	-1.5259883696	0.7252312745
H	-3.1986001081	-2.4859170089	1.0965592130
C	-1.4995361000	-1.4219300116	0.2917397360
H	4.3724363609	-1.1465157126	-1.7483304996
C	-0.5587788091	-2.5733279672	0.3076843357
H	-0.7553254853	-3.1926153208	-0.5820421407
H	0.4596461770	-2.1693126986	0.2645943325
H	-0.7274387796	-3.1818547315	1.2078377511
C	3.6294849743	-0.2873701440	0.1353768941
H	4.5168769255	0.0468887929	0.6518324180
N	2.2335104041	-0.8661975517	-1.5718739875
H	1.9936739720	-1.7130081830	-2.1005636238

ECI(1)

C	-0.1869416250	0.7022061102	0.0481793943
C	-1.5554859397	0.4815922237	-0.0856311629
C	-3.2342182515	-0.8076937309	-0.9287870272
C	-1.8487715719	-0.6255953920	0.9044297167
C	0.9780570701	-0.1804520099	-0.0661909103
C	2.1179340455	0.4940718534	0.4419351903
C	1.7852669184	1.8824832552	0.9084594021
C	0.2566579343	1.9453835956	0.7976600305
C	3.4783280339	-1.4058366564	-0.0467323236
C	3.3714048691	-0.1053057209	0.4558501239
C	-0.2515417462	3.1992851810	0.1172968182
O	-1.3531826806	-0.6781527530	2.0444530725
H	4.4562688638	-1.9047807452	-0.0395993403
H	2.2687525379	2.6238516743	0.2421357974
H	2.1358393803	2.0357485955	1.9454425907
H	-0.2191903745	1.8304823023	1.8032225442
H	-1.3333641743	3.1151614984	-0.0180356296
H	0.2313403086	3.3283584818	-0.8650669486
H	-0.0254459076	4.0770436127	0.7404596821
H	4.2440638927	0.4180048834	0.8478814754
C	2.3695976995	-2.0811643496	-0.5576216311
H	2.4904693849	-3.0989679900	-0.9400542385
C	1.0975744192	-1.4880660234	-0.5780359341
H	-4.0465784616	-1.0749292416	-1.5939137134
C	-0.0695964481	-2.2506978881	-1.1038730955
H	0.2703082625	-3.1513821399	-1.6400176837

H	-0.6937716048	-2.5489447923	-0.2572422960
H	-0.6526582359	-1.6255660829	-1.7836297643
C	-3.0201437049	-1.2910729778	0.3404320570
H	-3.4668046243	-2.1642428877	0.7966665034
N	-2.2914576770	0.1506237100	-1.2653400025
H	-2.6673335261	0.9783657061	-1.7447149746

ECI(2)

C	-0.2096525352	0.4239157833	-0.0839894233
C	-1.4007473556	-0.2467471545	-0.4541746827
C	-3.6370078311	0.1814560352	-0.7573781220
C	-2.1407681408	-0.4488965577	0.8641796835
C	1.0849378433	-0.2050799880	-0.1821842624
C	2.0636518825	0.5981074696	0.4613223829
C	1.4603551591	1.8702600795	0.9846272485
C	-0.0510981846	1.6828276657	0.7601779769
C	3.7094095339	-1.0654849102	-0.0150891560
C	3.3840935848	0.1808529629	0.5387260639
C	-0.6946086269	2.8625901222	0.0594192991
O	-1.6180650066	-0.8589153671	1.9114076403
H	4.7480324750	-1.4180957314	0.0479985045
H	1.8505329584	2.7327963910	0.4089001856
H	1.7056311066	2.0005915823	2.0551868468
H	-0.5714933462	1.4739502583	1.7261474689
H	-1.7460139829	2.6428076177	-0.1492729069
H	-0.1776277571	3.0745745816	-0.8894222683
H	-0.6306430320	3.7522263005	0.7036876801
H	4.1462608696	0.7919926059	1.0228747548
C	2.7527391511	-1.8706895111	-0.6339789591
H	3.0467915458	-2.8428627566	-1.0375223637
C	1.4143263770	-1.4599098449	-0.7386531722
H	-4.5472081424	0.3583715069	-1.3202610479
C	0.3662172710	-2.2972401849	-1.3832783968
H	-0.6120674625	-1.9039629414	-1.0850998229
H	0.4883227235	-2.2275848380	-2.4758362653
H	0.4903432590	-3.3459949145	-1.0778696537
C	-3.5531590986	-0.2995056689	0.5288908606
H	-4.3477110596	-0.3832276902	1.2535375688
N	-2.3838180658	0.3837768857	-1.3015835066
H	-2.2794052528	0.1639977777	-2.2968099759

6. Cartesian coordinates for three isomers of Filatov's two-stroke LDMRM DTPN optimized with the CAM-B3LYP/6-31G(d) method

Unit of the Cartesian coordinate below is angstrom.

(1) *EP* geometry at ground state.

C	1.29495100	-2.07075300	-0.67219500
C	-0.11258600	-1.88041700	-0.04312400
C	-0.30571200	-0.36080900	0.01465300
H	1.22670000	-2.27033300	-1.74859600
H	1.83984300	-2.90634000	-0.22138800
H	-0.89210200	-2.33619200	-0.65271400
C	-1.50479900	0.24471200	-0.00508800
C	-0.18666700	-2.49575100	1.35787800
H	0.56276400	-2.05251700	2.02288700
C	-1.83162500	1.71757200	-0.09693400
C	-3.28344000	1.74854000	-0.61740600
N	-3.78087800	0.43884300	-0.24713200
C	-2.79955000	-0.49594600	-0.04054000
O	-3.00606700	-1.69257600	0.09097100
C	3.04389200	1.37393200	0.09575900
S	3.54542900	-0.18744300	-0.47243900
C	1.92691700	-0.74497700	-0.40432300
C	1.04717200	0.22390900	-0.00212800
C	1.70362000	1.47158700	0.31617700
H	3.78485400	2.14274300	0.26952800
H	-1.17974800	-2.33781800	1.78129500
H	-0.00400200	-3.57426700	1.30905400
C	1.08941000	2.71178000	0.90108100
H	0.42096100	2.46846900	1.73198200
H	1.86984100	3.37367800	1.28486500
H	0.50994200	3.27961500	0.16815800
H	-4.71317600	0.12002700	-0.46628400
H	-3.87324300	2.54455700	-0.15446200
H	-3.31066400	1.89648700	-1.70554500
H	-1.78427300	2.20095700	0.88453500
H	-1.15937000	2.25846200	-0.76501000

(2) *ZP* geometry at ground state.

C	-1.50826600	2.04532900	-0.74478500
C	-0.11165300	2.07012200	-0.06460500
C	0.31212300	0.59838300	-0.03996300
H	-1.42198000	2.21430500	-1.82497400
H	-2.17967300	2.81249400	-0.34562800
H	0.59572100	2.66235600	-0.65309800
C	1.60491900	0.24665300	-0.12567500
C	-0.19976800	2.65712200	1.34849300
H	-0.93369800	2.10704600	1.94571200
C	2.77301800	1.21274300	-0.10726700
C	3.99442700	0.31180800	0.16112300
N	3.53087500	-0.98069500	-0.29753600
C	2.17026200	-1.10574300	-0.39290100
O	1.59423800	-2.13093800	-0.71368700
C	-2.77052900	-1.55228600	0.23233300
S	-3.48326300	-0.10892800	-0.42103500
C	-1.95009000	0.65136200	-0.43497900
C	-0.94153000	-0.17542000	-0.01400900
C	-1.42373700	-1.46861000	0.41658100
H	-3.40402400	-2.39390300	0.47759500
H	0.76222900	2.60167500	1.86634500
H	-0.50623000	3.70746900	1.31246500
C	-0.64155400	-2.55806700	1.08676200
H	0.00284900	-2.14507600	1.87024300
H	-1.32000000	-3.27810600	1.55249100
H	0.01129700	-3.07032700	0.38131000
H	4.11603300	-1.79764200	-0.38301100
H	4.88099000	0.63605800	-0.39100300
H	4.24883300	0.29506800	1.22960000
H	2.88533600	1.70530400	-1.08161800
H	2.67877200	1.99812200	0.64574300

(3) *ZM* geometry at ground state.

C	1.57204600	2.15808300	-0.21544200
C	0.14131800	2.07999100	0.39055800
C	-0.28036900	0.61834200	0.15487800
H	2.21981700	2.84600700	0.33663900
H	1.52069000	2.51911200	-1.25112900
H	0.24342100	2.22395200	1.47544900
C	-1.55133400	0.19648600	0.02850500

C	-0.75076000	3.18111000	-0.17617900
H	-0.98563000	2.99054900	-1.22844000
C	-2.82223800	0.95439500	0.36011700
C	-3.87697400	-0.16020700	0.51133300
N	-3.32882000	-1.21325600	-0.31566200
C	-1.98272800	-1.11187400	-0.54609600
O	-1.32799100	-1.91825600	-1.18375500
C	2.80290500	-1.54937900	0.18447400
S	3.54137000	-0.01841900	-0.17231500
C	2.00591200	0.73531400	-0.14467900
C	0.97988200	-0.14469000	0.06890500
C	1.44747700	-1.49406600	0.30370400
H	3.42707000	-2.42225300	0.31956400
H	-1.68859500	3.29552200	0.36856500
H	-0.22551000	4.14032300	-0.12055800
C	0.64448700	-2.68193000	0.74379100
H	0.09903600	-3.12590900	-0.08854400
H	1.29952900	-3.43301400	1.19389100
H	-0.09988300	-2.39129000	1.49269700
H	-3.82314500	-2.05267600	-0.57607100
H	-3.97547800	-0.47485400	1.55941700
H	-4.86369100	0.15185000	0.15830700
H	-2.74250100	1.55382200	1.26937500
H	-3.11913700	1.62296300	-0.45566100

7. References

[S1] W. Thiel, MNDO program, version 6.1, Max-Planck-Institut fur Kohlenforschung: Mulheim, 2007.

[S2] Frisch, M. J.; Trucks, G. W.; Schlegel, H. B.; Scuseria, G. E.; Robb, M. A.; Cheeseman, J. R.; Scalmani, G.; Barone, V.; Mennucci, B.; Petersson, G. A.; Nakatsuji, H.; Caricato, M.; Li, X.; Hratchian, H. P.; Izmaylov, A. F.; Bloino, J.; Zheng, G.; Sonnenberg, J. L.; Hada, M.; Ehara, M.; Toyota, K.; Fukuda, R.; Hasegawa, J.; Ishida, M.; Nakajima, T.; Honda, Y.; Kitao, O.; Nakai, N.; Vreven, T.; Montgomery Jr, J. A.; Peralta, J. E.; Ogliaro, F.; Bearpark, M.; Heyd, J. J.; Brothers, E.; Kudin, K. N.; Staroverov, V. N.; Keith, T.; Kobayashi, R.; Normand, J.; Raghavachari, K.; Rendell, A.; Burant, J. C.; Iyengar, S. S.; Tomasi, J.; Cossi, M.; Rega, N.; Millam, J. M.; Klene, M.; Knox, J. E.; Cross, J. B.; Bakken, V.; Adamo, C.; Jaramillo, J.; Gomperts, R.; Stratmann, R. E.; Yazyev, O.; Austin, A. J.; Cammi, R.; Pomelli, C.; Ochterski, J. W.; Martin, R. L.; Morokuma, R.; Zakrzewski, V. G.; Voth, G. A.; Salvador, P.; Dannenberg, J. J.; Dapprich, S.; Daniels, A. D.; Farkas, O.; Foresman, J. B.; Ortiz, J. V.; Cioslowski, J.; Fox, D. J.; Gaussian 09, Revision D.01, Gaussian, Inc., Wallingford CT, 2009.

[S3] Filatov, M.; Paolino, M.; Min, S. K.; Choi, C. H. Design and photoisomerization dynamics of a new family of synthetic 2-stroke light driven molecular rotary motors. *Chem. Commun*. **2019**, *55*, 5247-5250.
